# Correlation between tidal breathing pulmonary function, exhaled nitric oxide and airway hyperresponsiveness in children aged 0–3 years with suspected asthma

**DOI:** 10.3389/fped.2025.1388951

**Published:** 2025-07-25

**Authors:** Jiangjiao Qin, Fangjun Liu, Ting Wang, Zhou Fu, Ying Lin, Xia Wang, Jing Zhao, Sha Liu

**Affiliations:** ^1^ Department of Pulmonary Function, Children’s Hospital of Chongqing Medical University, Chongqing, China; ^2^ National Clinical Research Center for Child Health and Disorders, Ministry of Education Key Laboratory of Child Development and Disorders, Chongqing Key Laboratory of Pediatrics, Children’s Hospital of Chongqing Medical University, Chongqing, China; ^3^ Department of Respiratory Medicine, Children’s Hospital of Chongqing Medical University, Chongqing, China

**Keywords:** infants, pulmonary function, airway inflammation, FeNO, airway hyperresponsiveness (AHR)

## Abstract

**Objective:**

To investigate the correlation between tidal breathing pulmonary function parameters combined with mixed exhaled gas nitric oxide values and the degree of airway hyperresponsiveness (AHR) in children aged 0–3 years with suspected asthma.

**Methods:**

In this retrospective study, we collected baseline clinical data, tidal breathing pulmonary function parameters (measured before methacholine inhalation), fractional exhaled nitric oxide (FeNO) levels, and methacholine challenge test (MCT) results from 818 pediatric asthma patients treated at the Children's Hospital of Chongqing Medical University between January 2021 and June 2023. Baseline data, tidal respiratory pulmonary function parameters, and FeNO values were used to analyze their correlation with AHR. Ordinal multiclass logistic regression analysis was used to identify factors influencing AHR. The receiver operating characteristic (ROC) curve was performed to evaluate the efficacy of predicting AHR using tidal breathing pulmonary function parameters and FeNO values.

**Results:**

Intergroup comparisons showed significant differences in age, weight, height, FeNO, TPTEF/TE, RR, TI/TE, TEF50/TIF50, and PTFE/TEF25 (*P* < 0.05). Further ordinal multiclass logistic regression analysis revealed that increases in FeNO, RR, and PTEF/TEF25 were significantly positively correlated with AHR severity (*P* < 0.001), while age was significantly negatively correlated (*P* < 0.001). FeNO showed reasonable accuracy in predicting AHR at methacholine concentrations of 8 mg/ml (AUC=0.774) and a cut-off value of 14 ppb (sensitivity 88.5%, specificity 63.8%). The combined parameters (FeNO, RR, PTFE/TEF25, and age) showed high accuracy in predicting AHR at methacholine concentrations of 0.5 mg/ml (AUC=0.847).

**Conclusions:**

Our study revealed that Current airway inflammation and airway obstruction predicted AHR at this point.FeNO, RR, PTEF/TEF25, and age were effective predictive parameters for the degree of AHR in children aged 0–3 years with suspected asthma; FeNO >14 ppb served as an independent factor suggesting AHR in children at methacholine concentrations of 8 mg/ml, and the combined parameters showed better predictive efficacy.

## 1 Introduction

Airway hyperresponsiveness (AHR) is a pathological state of excessive airway responsiveness to stimuli associated with chronic airway inflammation and is one of the main features of asthma (1). The methacholine challenge test (MCT) is often used to quantify the degree of AHR and evaluate the patient's condition and treatment effect. Studies have shown that the level of airway reactivity in early infancy (1 month old) is strongly correlated with asthma in childhood (6 years old) (2, 3), and increased airway reactivity in childhood is an independent risk factor for asthma in adulthood (4, 5). Therefore, early identification of airway hyperresponsiveness in infancy is crucial to detect potential asthma risks in childhood, facilitate early intervention, and reduce the probability of developing asthma in adults. However, the clinical application of MCT is limited because of its complex operation, time-consuming nature, and high risk of inducing bronchospasm. Thus, it is urgent to find the predictive parameters of AHR.

Exhaled nitric oxide (fractional exhaled nitric oxide, FeNO) is commonly used in the clinic to evaluate airway inflammation in patients with asthma and the compliance and practicality of inhaled corticosteroid (ICS) treatment (5), thereby guiding the diagnosis and treatment of asthma. Additionally, tidal pulmonary function tests are significant in the severity assessment and prognosis of respiratory diseases in infants and young children. Previous studies have shown that tidal pulmonary function parameters in newborns can predict the severity of AHR and ICS usage at the age of ten (6). Scarce research has been conducted on the prediction of airway reactivity in infants and young children and is mostly limited to the prediction of positive or negative airway reactivity. Our study aimed to analyze the correlation between tidal pulmonary function parameters combined with FeNO and the degree of AHR to predict the degree of AHR in children aged 0–3 years with asthma and provide a reference for the choice of treatment methods and prognosis assessment.

## 2 Patients and methods

### 2.1 Patients

A retrospective analysis was performed on 818 children diagnosed with asthma from January 2021 to June 2023 in the Asthma Clinic of Children's Hospital of Chongqing Medical University. All included patients underwent the FeNO test, tidal breath pulmonary function test, and MCT on the day of consultation. The inclusion criteria were as follows: (1) Aged 0–3 years old, (2) Meeting the Global Initiative for Asthma (2023 edition) diagnostic criteria for asthma in children aged 5 and below to ensure that the children are in clinical remission; Specifically, symptoms of upper respiratory tract infection lasting more than 10 days, more than 3 exacerbations per year, having received ≥2 months of low-dose ICS treatment and showing clinical improvement. (3) Children were follow-up visits and had been diagnosed with asthma by a clinical doctor at least 2 times. (4) Excluding children with chest deformities, lower respiratory tract infection in the previous 4 weeks, or upper respiratory tract infection in the previous 1 week. This study was approved by the Research Ethics Committee of the Children's Hospital of Chongqing Medical University (Table 1).

**Table 1:** Demographic and functional parameter statistics and difference analysis of AHR degree.

Characteristic	Total *n* = 818	MCH (0.5 mg/ml) *n* = 34	MCH (2 mg/ml) *n* = 252	MCH (8 mg/ml) *n* = 394	MCH (16 mg/ml) *n* = 107	Non-responsive *n* = 31	*P*
Gender (Male)	505 (61.7)	22 (64.7)	153 (60.7)	243 (61.7)	67 (62.2)	20 (64.5)	0.984
Age (Month)	34.0 (26.0, 40.0)	23.5 (17.5, 34.0)	33.0 (24.0, 39.0)	35.0 (28.0, 40.0)	36.0 (29.0, 40.0)	39.0 (33.0, 41.0)	<0.001
Weight (kg)	14.0 (12.5, 15.0)	12.0 (10.5, 14.0)	14.0 (12.5, 15.0)	14.0 (12.5, 15.0)	14.0 (12.5, 15.5)	14.0 (13.0, 15.0)	0.001
Height (cm)	96.0 (90.0, 100.0)	87.5 (83.3, 97.0)	95.5 (89.0, 100.0)	96.0 (91.0, 100.0)	96.0 (92.0, 100.0)	99.0 (94.0, 102.0)	<0.001
FeNO (ppb)	27.0 (17.0, 39.0)	45.0 (26.5, 62.5)	32.0 (19.0, 43.0)	28.0 (20.0, 37.0)	11.0 (7.0, 20.0)	12.0 (5.0, 34.0)	<0.001
TPTEF/TE (%)	23.2 (19.5, 28.2)	23.3 (18.8, 27.1)	22.3 (19.3, 26.8)	23.0 (19.2, 28.4)	25.3 (20.5, 30.5)	24.1 (21.9, 30.9)	0.008
VPTEF/VE (%)	26.4 (23.9, 30.7)	25.6 (23.6, 29.7)	26.1 (23.8, 29.5)	26.4 (23.8, 30.8)	28.3 (24.3, 32.1)	28.1 (25.6, 33.9)	0.210
RR (pbm)	26.1 ± 4.3	28.9 ± 5.5	27.0 ± 4.1	25.4 ± 3.9	25.3 ± 4.9	25.5 ± 4.0	<0.001
VT/kg	8.9 (7.8, 9.8)	9.1 (7.4, 9.6)	8.7 (7.7, 9.8)	8.9 (7.8, 9.8)	8.9 (8.0, 9.9)	9.0 (7.4, 9.6)	0.544
tI/tE (%)	63.0 (56.0, 70.0)	62.5 (56.5, 71.0)	62.5 (55.0, 68.0)	63.0 (55.8, 69.0)	64.0 (58.0, 73.0)	68.2 (55.0, 77.0)	0.048
TEF50/TIF50 (%)	71.8 (63.3, 80.5)	68.4 (63.4, 78.8)	70.9 (62.1, 78.9)	72.5 (63.8, 80.7)	72.3 (62.8, 83.2)	74.0 (64.6, 90.4)	0.284
PTEF/TEF25 (%)	154.8 ± 25.3	162.6 ± 23.5	159.7 ± 25.2	153.4 ± 24.5	149.1 ± 26.1	144.4 ± 27.3	<0.001

Data are presented as Number (%), Median (P25, P75), or (x ± s).Comparison of Baseline Demographic Characteristics and Pre-Challenge Parameters Across AHR Severity Groups.

MCH(C%), methacholine concentration at the endpoint of methacholine challenge test; Non-responsive, methacholine challenge test methacholine concentration 16 mg/ml without an endpoint; FeNO, fractional exhaled nitric oxide; TPTEF/TE, time to reach peak tidal expiratory flow over expiratory time; VPTEF/VE, volume to reach peak tidal expiratory flow over expiratory volume; RR, respiratory rate; VT/kg, tidal volume per kilogram of body weight; TI/TE, the ratio of inspiratory time to expiratory time; TEF50/TIF50, tidal expiratory flow at 50% of expiratory volume divided by tidal inspiratory flow at 50% of inspiratory volume; PEF/TEF25, the ratio of peak expiratory flow rate to instantaneous expiratory flow rate with 25% tidal volume remaining.

### 2.2 Methods

Before all tests, children were induced into a stable sedated sleep state through oral administration of 10% chloral hydrate at a dosage of 30–50 mg/kg.

#### 2.2.1 FeNO measurement

FeNO was measured by offline tidal-breath NO measurement using a Sunvou nitric oxide analyzer (CA2123). Here are the operation steps: The appropriate tidal mask was selected and connected to the analyzer's special sampling bag through the tidal offline sampler (to filter NO in the air). The mask was tightly fastened to the subject's nose and mouth to prevent air leakage. After collecting more than 5 tidal breaths until the sampling bag was half full, the sampling bag was connected to the instrument for offline FeNO measurement, and the unit of the test result was ppb (parts per billion). FeNO was used by a technician specialized in pulmonary function tests before MCT, and the measurement precautions were strictly following the children's guidelines (5).

#### 2.2.2 Tidal breathing pulmonary function test

The tests were performed by a technician specialized in pulmonary function tests using a pulmonary function instrument (Jaeger MasterScreen Paed.CareFusion, San Diego, CA). The testing method strictly followed the quality control requirements of the Children's Tidal Breathing Pulmonary Function Guidelines (7).

#### 2.2.3 Methacholine challenge test

Before the test, the children's nasal secretions were cleared, and the transcutaneous arterial oxygen saturation (SPO_2_) of the children was monitored at the same time. Airway responsiveness can only be measured if at least one of the two parameters TPTEF/TE and VPTEF/VE exceeds 23% and SPO2 ≥ 95%.

Testing steps: Different concentrations of methacholine (MCH, Sigma, USA) solution were inhaled from low to high through the high-frequency atomizer by participants, with concentrations of 0.5, 2, 8, and 16 mg/ml in turn. Each atomization time was 1 min, and the inhalation time between different concentration intervals was 2 min. The tidal pulmonary function test was repeated 30–60 s after each aerosol inhalation of methacholine solution until the MCT was positive or the MCH concentration was the highest, and then the challenge process was terminated. Immediately after the stimulation process is terminated, a bronchodilator (1.25 mg terbutaline sulfate, 0.125 mg ipratropium bromide) combined with 4 ml of atomized inhalation solution is administered. SPO_2_, heart rate, and breathing were closely observed during the whole test. After 4–6 min, pulmonary function was retested, and the child's SPO_2_, heart rate, and pulmonary signs were evaluated until the parameters returned to the basic level.

Result determination: MCT-positive results were judged based on comprehensive indicators: ① Obvious expiratory wheezing appeared on lung auscultation; ② Respiratory frequency (RR) value increased by ≥50%; ③ SPO_2_ value decreased by ≥5. During the test, if any two of ①, ②, and ③ occur at the same time, it is considered an excitation endpoint. Degree classification: In the event of an excitation endpoint result, the degree was determined based on the concentration of inhaled MCH: 0.5, 2, 8, and 16 mg/ml, and no methacholine challenge test (MCT) endpoint at 16 mg/ml.

### 2.3 Statistical analysis

Data entry was performed using EXCEL, double entry, and check. Data analysis was performed using SPSS 27.0. Categorical variables were expressed as the number of cases (%), continuous variables conformed to a normal distribution and variance chi-square and were expressed as the mean ± standard deviation, and comparisons between multiple groups were performed using analysis of variance. Nonnormal continuous variables were expressed as M (P25, P75), and the Kruskal‒Wallis H test was used for comparisons between multiple groups. Correlations between parameters were analyzed using Spearman correlation analysis. Factors with statistically significant significance in the one-way analysis of factors were included in the multifactorial regression analysis as the independent variables, and factors with a strong correlation with age were excluded (height, body weight). The four concentrations (0.5 mg/ml, 2 mg/ml, 8 mg/ml, 16 mg/ml) at which the MCT showed provocation endpoints were assigned the values of 1, 2, 3, and 4, respectively, to represent different grades of airway responsiveness, and the ordered multiclassified logistic regression model was constructed, analyzing the independent influences on the degree of AHR. ROC curves were used to evaluate the efficacy of FeNO vs. the Combined parameter prediction at different degrees of AHR. Differences were considered statistically significant at *P* < 0.05.

## 3 Result

### 3.1 Characteristics of participants

A total of 7,957 children who completed tidal breathing pulmonary function test and MCT in the pulmonary function room of the Children's Hospital of Chongqing Medical University from January 2021 to June 2023 were selected, including 998 children who also completed FeNO on the same day. A total of 180 children whose diagnosis was unclear or who were not treated with ICS in outpatient medical records were excluded. A total of 818 children (aged 0–3 years, 505 males and 313 females) with asthma were included. Corresponding data of age, height, weight, and testing parameters were obtained. All participants were divided into 5 groups according to the severity of airway responsiveness determined by MCT. Comparisons of baseline characteristics and pre-challenge measurements (FeNO, tidal breathing parameters) among these groups are shown in Table 1. Statistically significant differences were observed in age, weight, height, FeNO, TPTEF/TE, RR, TI/TE, and PTFE/TEF25 (*P* < 0.05) (Table 1).

### 3.2 Correlation between age and parameters

There was a strong correlation between weight (*r* = 0.658, *P* < 0.001), height (*r* = 0.803, *P* < 0.001) and age. RR (*r* = −0.206, *P* < 0.001), TI/TE (*r* = 0.173, *P* < 0.001), TEF50/TIF50 (*r* = 0.232, *P* < 0.001), and AHR (*r* = 0.184, *P* < 0.001) were significantly related to age. There was no correlation between FeNO, TPTEF/TE, VPTEF/VT, VT/kg, PTEF/TEF25, and age (*P* > 0.05).

### 3.3 Correlation between pulmonary function parameters and AHR

Using assignment 4 (AHR at methacholine concentration at 16 mg/ml) as the reference category, ordered multicategorical logistic regression analysis was used (parallel validation significance level of 0.498, *P* > 0.05), and the model fit was *P* < 0.001. The results showed that an increase in FeNO, RR, and PTEF/TEF25 was significantly and positively correlated with the severity of AHR (*P* < 0.001)). An increase in age was negatively correlated with the severity of AHR (*P* < 0.001) (Table 2).

**Table 2:** Multiple ordinal logistic analysis of AHR.

Characteristic	B	*SE*	Wald *x*^2^	*P*	OR	OR 95%CI
FeNO (ppb)	−0.04	0.004	99.519	<0.001	0.962	0.954∼0.969
Age (month)	0.038	0.008	21.745	<0.001	1.039	1.022∼1.055
RR (pbm)	−0.093	0.016	32.071	<0.001	0.911	0.882∼0.941
TPTEF/TE (%)	0.007	0.012	0.33	0.566	1.007	0.984∼1.030
PTEF/TEF25 (%)	−0.013	0.004	11.684	0.001	0.987	0.979∼0.994

### 3.4 Efficiency value analysis of predicting AHR

**Figure 1:**
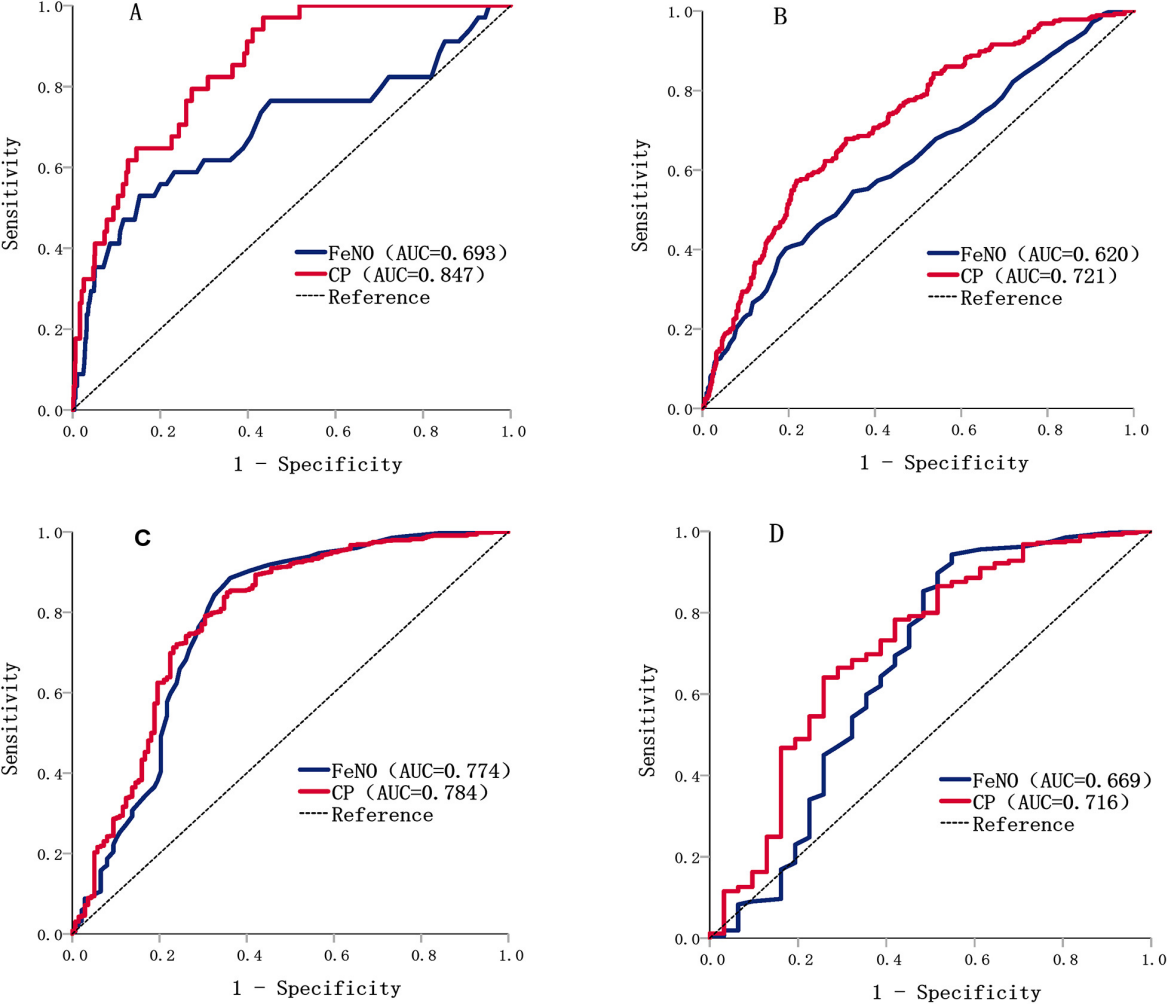
ROC curves of FeNO vs. combined parameters in predicting different degrees of AHR. **(A)** ROC curves of FeNO and combined parameters in predicting AHR at methacholine concentrations of 0.5 mg/ml; **(B)** ROC curves of FeNO and combined parameters in predicting AHR at methacholine concentrations of 2 mg/ml; **(C)** ROC curves of FeNO and combined parameters in predicting AHR at methacholine concentrations of 8 mg/ml; **(D)** ROC curves of FeNO and combined parameters in predicting AHR at methacholine concentrations of 16 mg/ml.

The ROC curve was used to evaluate the effectiveness of the independent parameter FeNO and the combined parameters (FeNO, RR, PTEF/TEF25, and age) in predicting different degrees of AHR (Table 3). The results showed that the accuracy of FeNO in predicting AHR at methacholine concentrations of 8 mg/ml was acceptable (AUC = 0.774), with a predictive threshold of 14 ppb. The accuracy of the combined parameters in predicting different degrees of AHR was acceptable and excellent in predicting AHR at methacholine concentrations of 0.5 mg/ml (AUC = 0.847) (Figure 1).

**Table 3:** Efficacy of feNO and combined parameters in predicting the corresponding AHR at different MCH concentrations.

Characteristic	MCH (C%)	AUC	95% CI of AUC	Sensitivity %	Specificity %	*P*
FeNO	0.5	0.693	0.661–0.725	52.94	84.69	<0.001
	2	0.620	0.586–0.653	40.21	80.64	<0.001
	8	0.774	0.774–0.802	88.53	63.77	<0.001
	16	0.669	0.635–0.701	94.28	45.16	0.001
Combined parameters	0.5	0.847	0.820–0.871	97.06	56.51	<0.001
	2	0.721	0.689–0.751	57.34	78.20	<0.001
	8	0.784	0.754–0.812	85.00	64.49	<0.001
	16	0.716	0.684–0.747	64.04	74.19	<0.001

Combined parameters, FeNO, RR, PTEF/TEF25, and age.

## 4 Discussion

Previous studies on the correlation between FeNO and age have mainly focused on children over 4 years old, and whether FeNO is strongly correlated with age is controversial (8, 9). Our results showed no significant correlation between FeNO and age in children under 4 years of age (*P* > 0.05). We found that AHR decreased with increasing age in infants and young children, which is consistent with previous studies (10).

Tidal breathing pulmonary function is an efficient and simple method for assessing airway obstruction in infants (11). TPTEF/TE and VPTEF/VE, which both have a high correlation, are crucial parameters indicating airway obstruction, with their decreasing values proportionally related to the severity of airway obstruction (7). However, TPTEF/TE is superior to VPTEF/VE in reflecting airway obstruction (12, 13). Our study found significant differences in TPTEF/TE with varying degrees of AHR (*P* < 0.05), whereas VPTEF/VE showed no significant variation across multiple groups, which confirmed the previous view. Geir Håland (6) demonstrated that reduced TPTEF/TE in the neonatal period is a significant predictor of severe AHR and the use of ICS at age 10. However, our study did not find a predictive value of TPTEF/TE for current AHR, suggesting that early airway conditions may be related to childhood AHR but not to current AHR. Morris (14) confirmed that the attenuation of flow over time during tidal breathing reflects respiratory system resistance. PTEF/TEF25 can quantify the rapid decline in flow during airway obstruction, and increased airway obstruction causes a decrease in TEF25 values and may lead to a shift from tidal to forced breathing, resulting in an increase in PTEF values. The inverse changes in these two parameters make the alterations in PTEF/TEF25 more pronounced, rendering it a sensitive indicator of increasing airway obstruction (15). Previous studies have also verified a high correlation between airway obstruction and AHR (16, 17), recommending FeNO combined with forced pulmonary function parameters of airways substituting for MCT in assessing AHR in asthma patients (18). Based on these known findings, our study showed significant differences in TPTEF/TE and PTEF/TEF25 across various groups, with a high correlation of PTEF/TEF25 with AHR. This suggested that airway obstruction may be the main cause of AHR in infants during asthma. Therefore, for this population, airway obstruction is a key factor of AHR, with its degree closely related to the severity of AHR. Our study highlighted the diagnostic and evaluative value of PTEF/TEF25 in infants and toddlers with asthma. Additionally, respiratory rate (RR) is a critical indicator for assessing children's physiological status and can be used for early warning scores of disease exacerbation (19). RR showed significant differences among children with varying degrees of airway reactivity, suggesting its potential as a predictive indicator for assessing the severity of asthma. Above all, it is recommended to pay special attention to the values of PTEF/TEF25 and RR when evaluating the effectiveness of asthma treatment in infants.

Tidal breathing NO measurement in infants offers the advantage of not requiring cooperation from the child and easy collection (20). However, as it involves collecting a mixed sample of oral and nasal gases, it may be affected by nasal NO (21). Previous research has indicated that the sinuses of infants are not fully developed and that tidal breathing methods are less affected by nasal NO (22), which has been confirmed by related studies (23). Online measurement of FeNO has been standardized, and offline methods have been proven applicable in infants (24). Bult (25) first functionally differentiated three subtypes of the enzyme system responsible for NO production in 1990, and activation of inducible NOS (iNOS) by airway inflammation might lead to increased NO production and thus escalating AHR (26). Relevant literature has also demonstrated that FeNO is related to AHR in asthma patients (27). Our study showed that FeNO >14 ppb could be used as an independent indicator of AHR with a sensitivity of 88.5% and a specificity of 63.8% when reacting to AHR with methacholine concentrations of 8 mg/ml. The combination of FeNO, RR, PTEF/TEF25, and age was a better predictor of AHR at methacholine concentrations of 0.5 mg/ml and 2 mg/ml than the independent parameter FeNO. It was reported that ventilation heterogeneity in asthmatic patients is determined by both inflammation and airway obstruction (28). PTEF/TEF25 could reflect the airway obstruction, which means that higher PTEF/TEF25 values indicate more severe obstruction. Meanwhile, the flow rate of FeNO depends on the airway diameter, which is positively correlated with the level of FeNO. Our results showed that the AUC of predicting AHR at methacholine concentrations of 0.5 mg/ml was 0.847. Thus, the combined parameters showed better predictive results in predicting stronger AHR, suggesting that as the severity of AHR increases, the influences on predicting the degree of AHR increase accordingly.

To clarify how this translates into clinical decision-making, we analysed dose-specific operating characteristics. At methacholine concentrations of 0.5 mg/ml the combined model is almost fail-safe for detecting strong airway hyper-responsiveness (sensitivity 97.06%), while FeNO alone is highly specific (84.69%). Thus the combined model is ideal for ruling out severe cases, with FeNO used afterwards to confirm truly positive children and avoid overtreatment.At methacholine concentrations of 16 mg/ml the pattern reverses: FeNO becomes the high-sensitivity screen (94.28%), and the combined model supplies the specificity needed (74.19%) to trim the large false-positive pool before starting inhaled corticosteroids or referral.This two-step approach pairs each model's strength, so clinicians minimise both missed diagnoses and unnecessary treatment.

This study is retrospective and shows some sample variation among groups, which could lead to bias due to unbalanced samples. However, the samples used in this study are consistent with the actual situation of this population. We did not include a saline control before the provocative process in our study, as doing so would not improve test safety but could reduce the accuracy of AHR measurements (29). Additionally, the inhalation time is shortened by increasing the MCH inhalation concentration (1 min), which has been proven feasible (30). We defined the study population as “suspected asthmatic children”, which was based on the GINA (2023 edition) criteria for the diagnosis of asthma at the age of 5 years and below and the diagnosis made by the clinicians. Although we used strict inclusion criteria, it is important to acknowledge that the diagnosis of asthma in children aged 0–3 years is controversial in the medical community, which constitutes a limitation of this study. Our study subjects were infants diagnosed with asthma and treated with ICS for more than 2 months, which is of great significance for monitoring and managing. Although there is no direct medical basis to support the hierarchical information that defines the four concentrations of 0.5/2/8/16 mg/ml as the degree of airway hyperresponsiveness, we have made this assumption based on a general understanding of airway responsiveness and the physiological effects that may result from changes in concentration. We acknowledge the limitations of this approach and view it as a preliminary exploration. Future studies should further validate the reasonableness of this hierarchy.

In conclusion, airway inflammation and airway obstruction were strongly associated with the degree of AHR and were responsible for the development of AHR in infants with suspected asthma during the remission period. Meanwhile, the effect of age on AHR needs to be calibrated in interpreting MCT results in this age range. FeNO, RR, PTFE/TEF25, and age as combined parameters have certain accuracy in predicting different degrees of AHR. Therefore, the noninvasive airway inflammation marker FeNO combined with baseline tidal breathing pulmonary function parameters is valuable in predicting the degree of AHR.

## Data Availability

The original contributions presented in the study are included in the article/Supplementary Material, further inquiries can be directed to the corresponding author.
